# Genome-Wide Identification of the *FWL* Gene Family in Rice Reveals Critical Roles in Abiotic Stress Response

**DOI:** 10.3390/plants15081146

**Published:** 2026-04-08

**Authors:** Xuefei Ma, Yi Ji, Minghao Wang, Linlin Liu, Fanhao Nie, Xin Meng, Juan Zhao, Qingpo Liu

**Affiliations:** College of Advanced Agricultural Sciences, Zhejiang A&F University, Lin’an, Hangzhou 311300, China

**Keywords:** Fruit Weight 2.2-like (FWL), abiotic stress, arsenic tolerance, *OsFWL8*, rice

## Abstract

The Fruit Weight 2.2-like (*FWL*) gene family, characterized by the conserved PLAC8 domain, plays important roles in plant organ development and metal ion homeostasis. However, the systematic characterization of *FWL* genes in rice (*Oryza sativa*) and their involvement in abiotic stress responses remain insufficiently understood. In this study, a genome-wide identification of the *FWL* gene family in rice was performed, resulting in the identification of nine *OsFWL* genes, including a previously unreported member, *OsFWL9*. Phylogenetic analysis of FWL proteins from rice, maize, soybean, and *Arabidopsis thaliana* classified the family into three distinct subgroups, indicating both conserved and divergent evolutionary relationships. Structural and conserved motif analyses revealed that OsFWL proteins share similar domain architectures, while promoter analysis uncovered abundant *cis*-acting elements associated with stress responses, phytohormone signaling, and plant growth and development. Expression profiling demonstrated that most *OsFWL* genes were rapidly induced by drought, high temperature, salt, and arsenic stresses at the seedling stage, suggesting their broad involvement in abiotic stress adaptation. Notably, *OsFWL8* exhibited a unique expression pattern, being significantly suppressed under arsenic stress. Functional characterization using CRISPR/Cas9-generated knockout mutants and overexpression lines revealed that *OsFWL8* negatively regulates arsenic tolerance in rice. Overexpression of *OsFWL8* markedly increased plant sensitivity to arsenic stress. Furthermore, arsenic detoxification-related genes, including *OsABCC1* and *OsPCS2*, were significantly upregulated in *fwl8* mutants under arsenic treatment. These results indicate that *OsFWL8* may modulate arsenic tolerance by influencing arsenic sequestration and detoxification pathways. Overall, this study provides a comprehensive overview of the *FWL* gene family in rice and identifies *OsFWL8* as a key regulator of arsenic stress response, offering valuable insights for improving rice tolerance to heavy metal stress.

## 1. Introduction

Abiotic stresses, including high temperature, drought, salinity, and heavy metal toxicity, are major environmental constraints that severely limit plant growth, development, and crop productivity [1,2]. During long-term evolution, plants have developed sophisticated physiological and biochemical mechanisms that enable them to perceive and respond rapidly to adverse environmental conditions. These adaptive responses include the synthesis of stress-responsive proteins, accumulation of osmolytes, activation of antioxidant defense systems, modulation of phytohormone signaling, and maintenance of cellular ion homeostasis. Collectively, these processes help alleviate stress-induced damage such as reduced cellular osmotic potential, membrane destabilization, altered membrane fluidity, and intracellular solute imbalance [3].

Plant stress tolerance is a highly complex trait involving polygenic regulation and the coordination of multiple signaling pathways. These responses are mediated by intricate intracellular networks composed of protein–protein interactions and signal transduction cascade [4]. In rice, for example, the salt tolerance gene *OsRST1* negatively regulates the expression of *OsAS1*, an asparagine synthetase gene, thereby restricting asparagine biosynthesis and leading to excessive ammonium (NH_4_^+^) accumulation, which compromises nitrogen utilization efficiency. During rice domestication, RST1 underwent directional selection, and the superior haplotype *RST1* Hap III exhibits attenuated repression of *OsAS1*, resulting in enhanced salt tolerance [5]. In addition, maintenance of cellular Na^+^/K^+^ homeostasis is essential for plant adaptation to saline–alkali stress [6]. The plasma membrane Na^+^/H^+^ antiporter *OsSOS1* plays a critical role in regulating Na^+^ efflux from rice stems, thereby sustaining a low intracellular Na^+^/K^+^ ratio and improving salt tolerance [7]. Reducing the accumulation of reactive oxygen species is also one of the strategies for plants to resist abiotic stress. Under salt stress, the *OsRST3* mutant reduces DeoxyriboNucleic Acid (DNA) damage and the accumulation of reactive oxygen species, thereby exhibiting tolerance. The absence of *OsRST31* blocks the transport of cytokinin from the roots to the leaves; under normal conditions, high levels of cytokinin in the leaves exacerbate the accumulation of reactive oxygen species, thereby reducing rice salt tolerance [8]. For plants, the cell wall serves as an external barrier against stress. Salt stress induces the expression of *OsCSLD4*, a gene involved in cell wall hemicellulose synthesis. Studies have shown that when *OsCSLD4* function is lost, hemicellulose accumulation in the cell wall decreases and cell wall integrity is compromised, leading to a significant decline in rice plants’ tolerance to salt stress [9].

High temperature can severely impact pollen viability, grain setting, and the grain-filling process, leading to a significant yield penalty. Key mechanisms such as enhancing ROS (reactive oxygen species) scavenging, synthesizing osmoregulatory substances, and regulating transcription factors play important roles in the defense against heat stress. In rice, members of the NAC, MYB, and WRKY families have been widely reported to participate in heat stress responses. NAC proteins are plant-specific transcription factors involved in diverse aspects of development and stress adaptation [10]. *OsNAC127* and *OsNAC129* are heat-responsive genes that play essential roles in grain filling by regulating sugar transport and stress-responsive gene expression; knockout of either gene leads to impaired grain filling, particularly under high-temperature conditions [11]. Similarly, the R2R3-MYB transcription factor OsMYB55 is induced by heat stress, and its overexpression significantly enhances rice growth and grain yield under elevated temperature [12].

Drought stress profoundly affects plant root architecture, including root length, root number, and lateral root development, thereby influencing water acquisition capacity. Upon drought perception, phytohormones such as abscisic acid (ABA) trigger transcriptional reprogramming that modulates root development and improves water uptake. The transcription factor OsbHLH120, which is induced by ABA and polyethylene glycol (PEG), enhances drought tolerance by increasing root thickness and length [13,14]. In addition, *OsMADS23* and *OsbZIP86* positively regulate drought resistance in rice. ABA acts as a key signaling molecule in drought responses by inducing stomatal closure through complex regulatory networks, thereby reducing transpirational water loss and improving water use efficiency. *OsMADS23* and *OsbZIP86* are phosphorylated by the upstream kinases *OsSAPK9* and *OsSAPK10*, respectively, promoting ABA accumulation and stomatal closure and ultimately enhancing drought tolerance [15].

Heavy metal stress also poses a serious threat to rice production and food safety. In rice, efforts to enhance arsenic stress tolerance have focused on reducing arsenic accumulation in rice grains. Arsenic transport primarily occurs through root uptake and translocation to the aboveground parts; the genes *OsPht1;9* and *OsPht1;10* encode a phosphate transporter primarily responsible for the uptake of arsand its transport to the aboveground parts. Simultaneous knockout of these two genes significantly reduces arsenic content in grains without affecting yield [16]; *OsPIP2;6* encodes an intrinsic plasma membrane protein localized in the vascular tissues of roots and leaves, responsible for the transport of arsenic from the roots to the aboveground parts. Studies have shown that inhibiting the expression of *OsPIP2;6* reduces arsenic content in rice shoots by 35–65% [17]. The ABC transporter OsABCC1 plays a pivotal role in detoxifying arsenic by transporting As–PC complexes into vacuoles, and loss of *OsABCC1* function results in increased arsenic sensitivity [18]. In contrast, suppression of *OsPCS1* and *OsPCS2* reduces cadmium and arsenic accumulation, while overexpression of *OsWNK9* and *OsPRX38* enhances arsenic tolerance in rice [19,20]. Despite these advances, the identification and functional characterization of additional rice genes involved in abiotic stress responses remain essential for the development of stress-resilient cultivars.

The Fruit-Weight-like (*FWL*) gene family, characterized by a cysteine-rich PLAC8 domain, comprises proteins implicated in the regulation of grain size, metal ion homeostasis, and organ development. In rice, the *OsFWL* family represents an important group of genes that modulate cell size and organ growth. This family was initially identified through bioinformatic analyses based on sequence homology to the tomato *FW2.2* gene [21]. FWL proteins are membrane-associated and participate in the control of cell division and expansion, thereby influencing agronomic traits such as grain size, yield, and stress tolerance [22]. In *Zea mays*, *ZmCNR1* and *ZmCNR2* regulate cob size, while in *Arabidopsis thaliana*, *AtPCR1/AtFWL7* enhances cadmium tolerance [23]. Another family member, *AtPCR2/AtFWL3*, functions as a zinc efflux transporter and is essential for maintaining zinc homeostasis under fluctuating environmental conditions [24].

In rice, several *FWL* genes have been associated with grain morphology and metal transport. Mutations in *OsFWL4* reduce cadmium translocation from roots to shoots, thereby enhancing Cd tolerance, whereas *OsFWL3*, *OsFWL6*, and *OsFWL7* confer cadmium tolerance in yeast systems. However, the biological function of *OsFWL8* remains largely unexplored [25,26]. *OsFWL5*/*OsPCR1* positively regulates grain weight and zinc accumulation while simultaneously reducing cadmium content in rice [27], and *OsFWL1*/*OsTGW2* has been identified as a negative regulator of grain width [28]. Despite these findings, the roles of the *FWL* gene family in rice abiotic stress responses remain poorly understood.

In this study, we systematically identified FWL-encoding genes in rice using bioinformatic approaches and analyzed their evolutionary relationships, gene structures, and *cis*-regulatory elements. Furthermore, we investigated the responses of *OsFWLs* to abiotic stresses and elucidated the physiological function of *OsFWL8* in arsenic tolerance through overexpression and CRISPR/Cas9-mediated gene editing. Collectively, our results expand current knowledge of the rice *FWL* gene family and provide a theoretical foundation for future studies aimed at improving abiotic stress tolerance in rice.

## 2. Materials and Methods

### 2.1. Identification of the FWL Gene Family

The Hidden Markov Model (HMM) profile of the FWL protein which contains the PLAC8 conserved domain (Pfam accession number: PF04749) was retrieved from the Pfam database. Candidate *FWL* genes were identified from the complete protein sequences of the rice (*Oryza sativa*) genome using HMMER v3.1 software. Annotation information for the selected *FWL* family members was obtained from RiceData (Rice Genome Annotation Project). The *FWL* gene family members of *Arabidopsis thaliana*, *Glycine max*, and *Zea mays* were obtained from the Phytozome database (https://phytozome.jgi.doe.gov/ (accessed on 3 February 2026)) [29]. Basic information on *FWL* gene family members in *Oryza sativa*, *Arabidopsis thaliana*, *Glycine max*, and *Zea mays* is provided in Supplemental Table S1.

### 2.2. Phylogenetic Analysis of FWL Genes

*FWL* family members were identified by BLASTP (https://blast.ncbi.nlm.nih.gov/Blast.cgi?PROGRAM=blastp&PAGE_TYPE=BlastSearch&LINK_LOC=blasthome (accessed on 3 February 2026)) searches against the genomes of four plant species, including *Oryza sativa*, *Arabidopsis thaliana*, *Glycine max*, and *Zea mays* [30]. Align the 47 FWL protein sequences using the MUSCLE tool in the MEGA11 (Version 11.0.13) software. After the alignment is complete, construct a tree using the maximum likelihood method and save the Newick tree file. The phylogenetic tree was visualized and annotated using the Interactive Tree of Life (iTOL) web tool (https://itol.embl.de/ (accessed on 3 February 2026)).

### 2.3. Analysis of FWL Gene Structures

Gene structure analysis of *FWL* genes was performed using the GFF3/GTF (Rice Genome Annotation Project, https://rice.uga.edu/download_osa1r7.shtml accessed on 3 February 2026)) annotation files of *Oryza sativa*. Exon–intron organization was extracted and visualized using the Gene Structure View (Advanced) module in TBtools (V2.450). Conserved motifs were predicted using the MEME Suite, and conserved domains were identified through the NCBI Conserved Domain Database (CDD). All results were visualized using TBtools (V2.450) software [31].

### 2.4. Analysis of Cis-Acting Elements in Gene Promoters

Potential promoter sequences spanning 2000 bp upstream of the ATG start codon of each *OsFWL* gene were extracted using TBtools v2.041. *Cis*-acting regulatory elements within these promoter regions were identified using the PlantCARE database (https://bioinformatics.psb.ugent.be/webtools/plantcare/html/ (accessed on 5 February 2026)). The distribution and classification of *cis*-elements were visualized using TBtools to generate corresponding charts.

### 2.5. Plant Materials and Arsenic Treatment

The *japonica* rice cultivar Nipponbare was used as the wild-type (WT) control. Transgenic rice lines overexpressing *OsFWL8* (OE-*OsFWL8*) and *OsFWL8* knockout lines (*fwl8*), both in the Nipponbare background, were used for subsequent analyses. Rice seeds were germinated for two days and then treated with 25 μM As(III) for seven days. In addition, uniformly grown 21-day-old WT and transgenic seedlings were subjected to a 3-day treatment with or without 25 μM As(III), following previously described methods [32].

### 2.6. Vector Construction and Genetic Transformation

Gene-specific primers were designed to amplify the coding sequence (CDS) of *OsFWL8* (*LOC_Os03g61480*) from the Nipponbare genome. The amplified CDS was cloned into the pCAMBIA1300-GFP vector to generate the *35S*::*OsFWL8*::GFP construct. The *fwl8* mutant line was generated using CRISPR/Cas9-mediated genome editing. All constructs were introduced into the Nipponbare background via *Agrobacterium tumefaciens*-mediated transformation to obtain transgenic rice lines [33]. Primer sequences used for vector construction are listed in Supplemental Table S2.

### 2.7. Stress Treatments and Experimental Conditions

For high-temperature treatment, rice seedlings were grown under normal conditions until 14 days old and then transferred to a growth chamber maintained at 45 °C [34]. For drought treatment, 14-day-old seedlings were transferred to nutrient solution supplemented with 20% polyethylene glycol (PEG) 6000 [35]. Salt stress was imposed by transferring 14-day-old seedlings to nutrient solution containing 150 mM NaCl [36,37]. For arsenic treatment, 21-day-old seedlings were transferred to nutrient solution containing 40 μM As(III) [32]. Samples were collected at designated time points (0 h, 1 h, 3 h, and 6 h), immediately frozen in liquid nitrogen, and stored at −80 °C for subsequent RNA extraction and quantitative real-time PCR (qRT-PCR) analysis.

### 2.8. Quantitative Real-Time PCR Analysis

Total RNA was extracted from rice samples using the Plant RNA Easy Fast Kit (TIANGEN, China). Following DNase I treatment, first-strand cDNA was synthesized using the Hifair III 1st Strand cDNA Synthesis Super Mix (YEASEN, China). Quantitative real-time PCR was performed using gene-specific primers and Hieff qPCR SYBR Green Master Mix (No Rox) (YEASEN, China) on a Bio-Rad CFX96 Real-Time PCR System (Bio-Rad, USA). The PCR program consisted of an initial denaturation at 95 °C for 5 min, followed by 40 cycles of 95 °C for 15 s, 60 °C for 30 s, and 72 °C for 30 s. The rice ubiquitin gene *OsUBQ5* (*LOC_Os01g22490*) was used as the internal reference, and relative gene expression levels were calculated using the 2^−ΔΔCT^ method [38]. Primer sequences used for qRT-PCR are listed in Supplemental Table S2.

## 3. Results

### 3.1. Identification and Evolutionary Analysis of FWL Genes

The Hidden Markov Model (HMM) profile of the FWL protein signature domain was retrieved from the Pfam database (ID: PF04749). Using HMMER v3.1, candidate *FWL* genes were identified from the genome-wide protein sequences of rice and further validated through comparison with *FWL* family members in *Arabidopsis thaliana*. Ultimately, nine *FWL* genes were identified in the rice genome (Table S1).

**Figure 1:**
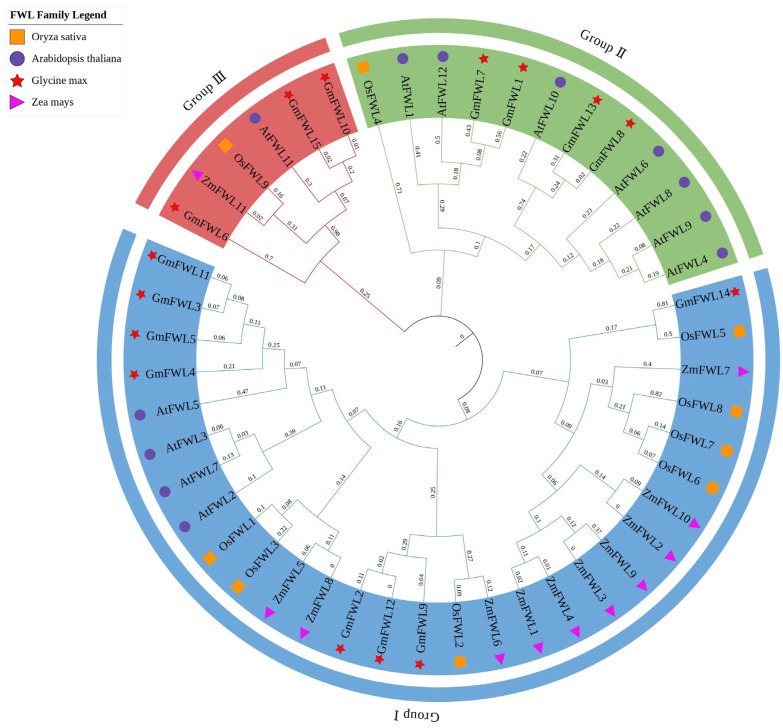
Phylogenetic analysis of FWL proteins in rice, soybean, maize, and *Arabidopsis*. A phylogenetic tree was constructed using iTOL to illustrate the evolutionary relationships among 47 FWL family members from rice (*Oryza sativa*), soybean (*Glycine max*), maize (*Zea mays*), and *Arabidopsis thaliana*. The tree divides the FWL proteins into three distinct phylogenetic subfamilies (I–III), which are indicated by different colors.

To investigate the evolutionary relationships of FWL proteins among different plant species, *FWL* family members from four representative species—rice (*Oryza sativa*), maize (*Zea mays*), soybean (*Glycine max*), and *Arabidopsis thaliana*—were analyzed, and a phylogenetic tree was constructed based on their protein sequences (Figure 1). Phylogenetic analysis classified the FWL proteins into three distinct subgroups. Group I contained multiple members from all four species, suggesting that genes within this clade are evolutionarily conserved and may represent a common adaptive strategy that enables functional diversification across plant lineages. Within this subgroup, *OsFWL6*, *OsFWL7*, and *OsFWL8* clustered closely together, indicating a strong paralogous relationship that likely arose from local gene duplication events. Group II includes multiple genes from *Arabidopsis* and soybean, as well as the *OsFWL4* gene; however, *OsFWL4* is distantly branched from the other genes, which may reflect specific evolutionary differences between dicotyledons and monocotyledons. Group III includes genes from all four species but contains fewer genes, comprising only *OsFWL9*, *ZmFWL11*, *AtFWL11*, *GmFWL6*, *GmFWL10*, and *GmFWL15*. Among these, *OsFWL9* is the only gene in the rice FWL family that possesses a unique structural domain, suggesting it may have undergone specialized functional differentiation. This phylogenetic tree reveals that rice and maize FWL proteins exhibit closer evolutionary relationships on certain branches of the phylogenetic tree. Meanwhile, the clustering of specific *Arabidopsis* and soybean *FWL* members (e.g., *AtFWL10* and *GmFWL13*) reflects conserved evolutionary relationships within dicotyledonous species.

### 3.2. Conserved Motifs, Domains, and Gene Structures of OsFWLs

**Figure 2:**
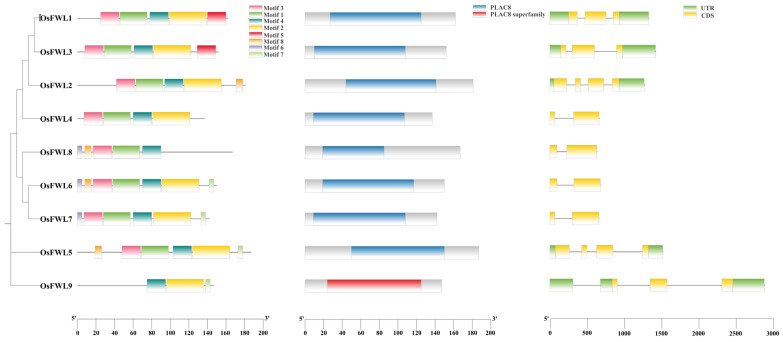
Conserved motifs, conserved domains, and gene structures of OsFWLs. (**Left**) Distribution of conserved motifs predicted by MEME, with different motifs indicated by distinct colors. (**Middle**) Conserved domain composition identified using the NCBI CDD database. (**Right**) Gene structures of *OsFWL* genes, showing CDS and UTR regions.

Using rice GFF3/GTF annotation files, we systematically analyzed the gene structures of all members of the *OsFWL* gene family. Conserved motifs in OsFWL proteins were predicted using the MEME online tool, and conserved domains were identified using the NCBI Conserved Domain Database (CDD). A comprehensive diagram integrating conserved motifs, conserved domains, and gene structures of *OsFWL* family members was generated (Figure 2). Analysis of conserved motifs showed that each *OsFWL* gene encodes between three and seven conserved motifs, with most genes containing five or six motifs (Figure 2). These motifs correspond to conserved structural elements within the PLAC8 domain. Domain architecture analysis revealed that OsFWL9 contains only the PLAC8 superfamily domain, whereas the remaining eight *OsFWLs* possess the canonical PLAC8 domain, indicating pronounced structural divergence between OsFWL9 and OsFWL1–OsFWL8 (Figure 2). Consistent with phylogenetic relationships, genes clustered within the same clade displayed similar domain organization and motif distribution, exemplified by *OsFWL6*, *OsFWL7*, and *OsFWL8*. Gene structure analysis further showed that rice *FWL* genes contain two to four coding sequences (CDSs). Notably, *OsFWL4*, *OsFWL6*, *OsFWL7*, and *OsFWL8* lack both upstream and downstream untranslated regions (UTRs) (Figure 2).

### 3.3. Cis-Acting Element Analysis of OsFWL Gene Promoters

**Figure 3:**
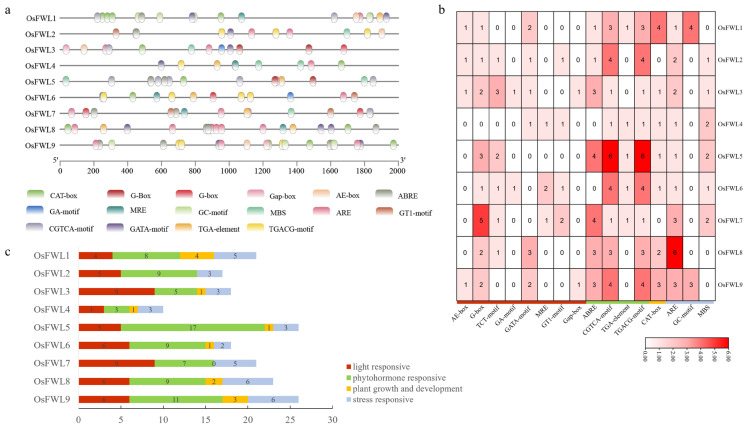
*Cis*-acting element analysis of OsFWL promoters. (**a**) Distribution of predicted *cis*-acting elements within the 2000 bp promoter regions upstream of the ATG start codon. (**b**) Quantitative statistics of different *cis*-element types in *OsFWL* promoters. (**c**) Classification of *cis*-elements into light-responsive (red), phytohormone-responsive (green), growth and development-related (yellow), and stress-responsive (blue) categories.

Promoter sequences spanning 2000 bp upstream of the ATG start codon of each *OsFWL* gene were retrieved from the NCBI database. *Cis*-acting regulatory elements within these promoter regions were identified using the PlantCARE database (Figure 3a–c). The results revealed that *OsFWL* promoters harbor abundant light-responsive elements (e.g., AE-box, G-box, TCT-motif), phytohormone-responsive elements (e.g., ABRE, CGTCA-motif, TGA-element), growth- and development-related elements (e.g., CAT-box), and stress-responsive elements (e.g., ARE, MBS) (Figure 3a). Specifically, the promoter of *OsFWL1* contained the highest number of CAT-box and GC-motif elements. *OsFWL2*, *OsFWL6*, and *OsFWL9* each harbored four CGTCA-motif and TGACG-motif elements, while *OsFWL5* contained six such elements. The *OsFWL7* promoter was enriched with G-box and ABRE elements, and *OsFWL8* possessed the highest number of ARE elements. Classification of the predicted *cis*-elements indicated that *OsFWL3* and *OsFWL7* promoters were enriched in light-responsive elements, *OsFWL5* showed the highest abundance of hormone-responsive elements, *OsFWL1* contained the most growth- and development-related elements, and *OsFWL8* and *OsFWL9* were enriched in stress-responsive elements (Figure 3b,c). These results suggest that *OsFWL* genes may play important roles in rice growth, hormonal regulation, and abiotic stress responses.

### 3.4. Expression Responses of OsFWL Genes to High-Temperature Stress

**Figure 4:**
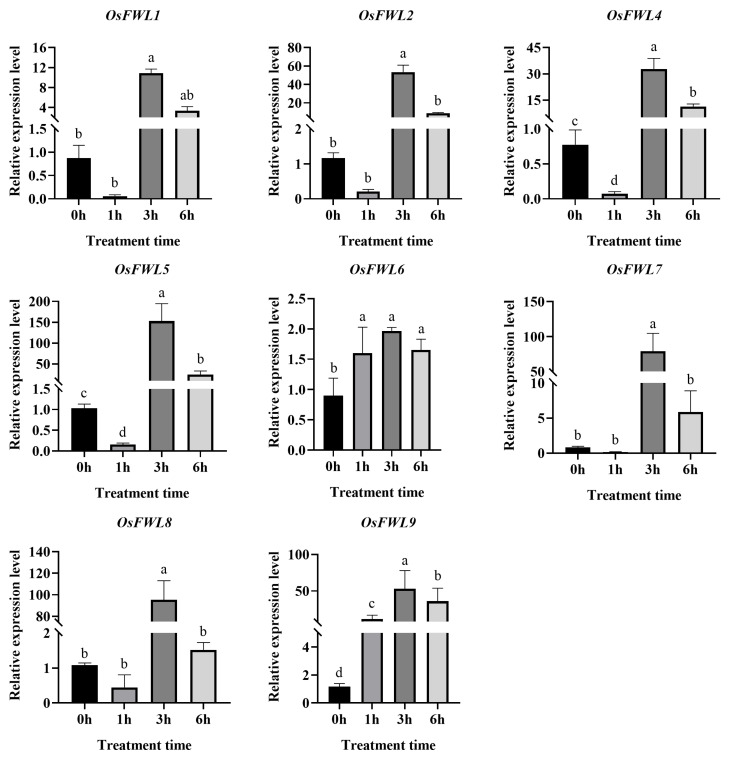
Expression profiles of *OsFWL* genes under heat stress. Expression levels were measured by qRT-PCR. Data represent mean ± SD (*n* = 3). Different letters indicate significant differences (*p* < 0.05).

Fourteen-day-old Nipponbare seedlings were subjected to 45 °C heat stress, and gene expression patterns were examined by qRT-PCR (Figure 4). Unexpectedly, *OsFWL3* exhibited extremely low expression levels in seedlings (Figure S1). Tissue-specific expression analysis further confirmed that OsFWL3 was weakly expressed across rice tissues; therefore, subsequent analyses focused on *OsFWL1*, *OsFWL2*, *OsFWL4*, *OsFWL5*, *OsFWL6*, *OsFWL7*, *OsFWL8* and *OsFWL9*. Under high-temperature treatment, all eight genes were significantly induced, with expression levels peaking at 3 h post-treatment. Among them, *OsFWL2*, *OsFWL7* and *OsFWL9* showed strong induction, each increasing by approximately 50-fold. Furthermore, *OsFWL5* and *OsFWL8* demonstrated particularly strong induction, increasing by approximately 150-fold and 100-fold respectively. These results indicate that multiple *OsFWL* genes respond positively to heat stress, with *OsFWL5* and *OsFWL8* exhibiting the strongest responses.

### 3.5. Expression Responses of OsFWL Genes to Drought Stress

**Figure 5:**
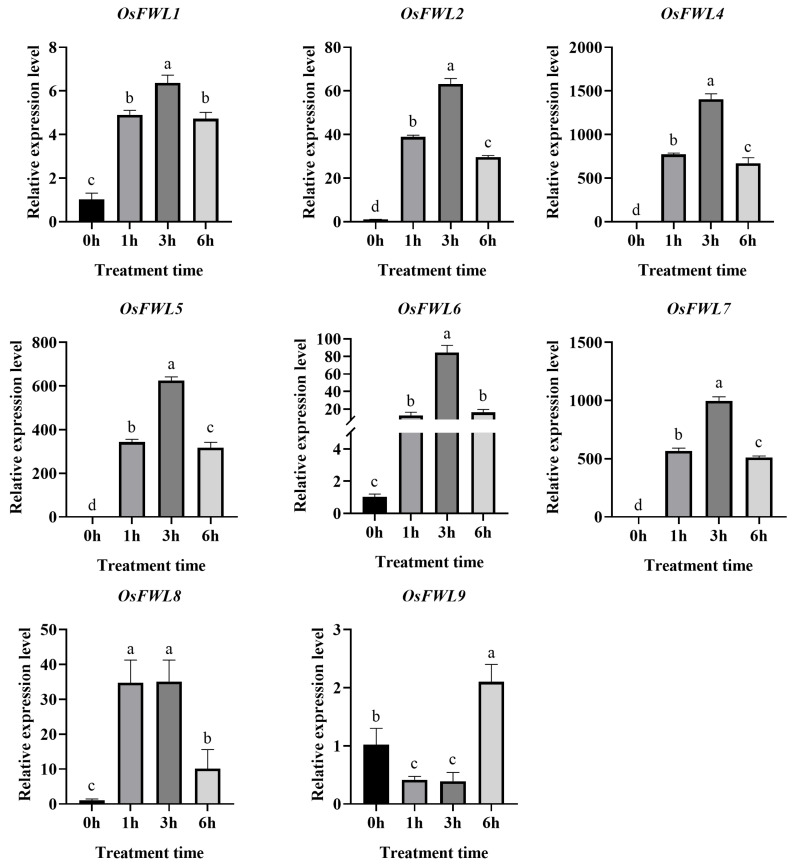
Expression profiles of *OsFWL* genes under drought stress. Data are presented as mean ± SD (*n* = 3). Different letters indicate significant differences (*p* < 0.05).

To investigate the involvement of *FWL* genes in drought responses, drought stress was simulated using 20% PEG 6000 (Figure 5). Expression levels of *OsFWL1*, *OsFWL2*, *OsFWL4*-*8* were significantly induced as early as 1 h after treatment, reaching peak levels at 3 h. Notably, *OsFWL4*, *OsFWL5*, and *OsFWL7* showed exceptionally strong responses, with expression levels exceeding 200-fold at 1 h and further increasing to over 600-fold at 3 h. Expression of *OsFWL5* and *OsFWL7* reached nearly 1000-fold induction, indicating that these genes are highly sensitive to drought stress. Interestingly, the expression level of the *OsFWL9* gene was significantly suppressed in the early stage of treatment, but it increased significantly and reached its peak after 6 h of treatment. These findings suggest that *FWL* family genes play important roles during the early stages of drought response in rice.

### 3.6. Expression Responses of OsFWL Genes to Salt Stress

**Figure 6:**
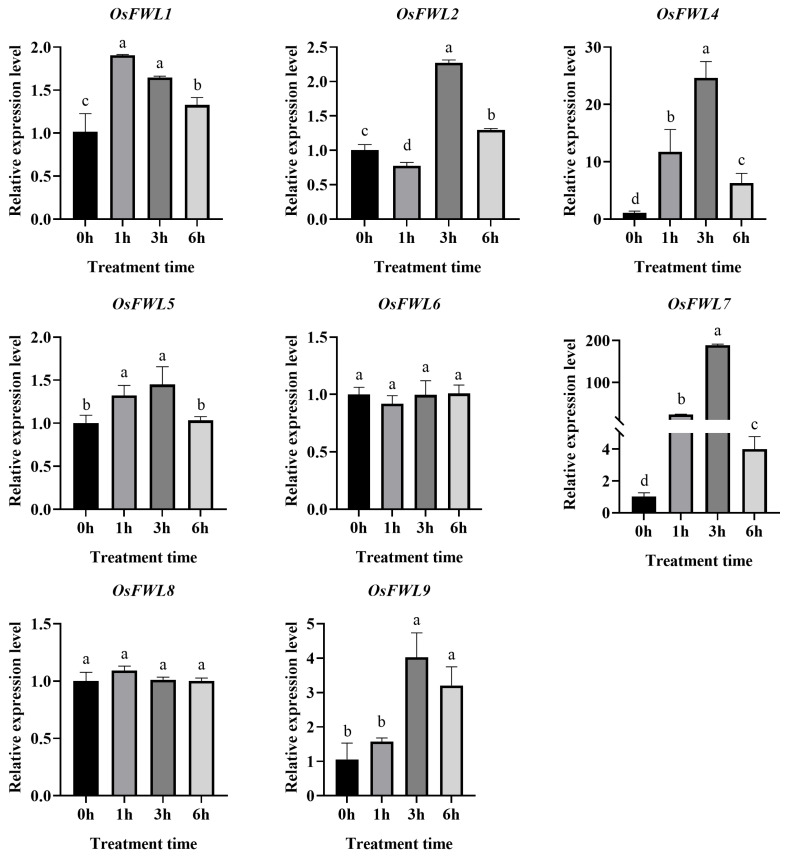
Expression profiles of *OsFWL* genes under salt stress. Data are presented as mean ± SD (*n* = 3). Different letters indicate significant differences (*p* < 0.05).

To examine salt stress responses, 14-day-old Nipponbare seedlings were treated with 150 mM NaCl, and expression levels of selected *OsFWL* genes were monitored by qRT-PCR (Figure 6). Except for *OsFWL6* and *OsFWL8*, all analyzed genes were significantly induced by salt stress, displaying an initial increase followed by a gradual decline. *OsFWL1* reached peak expression at 1 h post-treatment, whereas *OsFWL7* exhibited a strong induction of approximately 200-fold at 3 h. These results indicate that most *OsFWL* genes are activated during the early phase of salt stress response.

### 3.7. Responses of OsFWL Genes to Arsenic Stress

**Figure 7:**
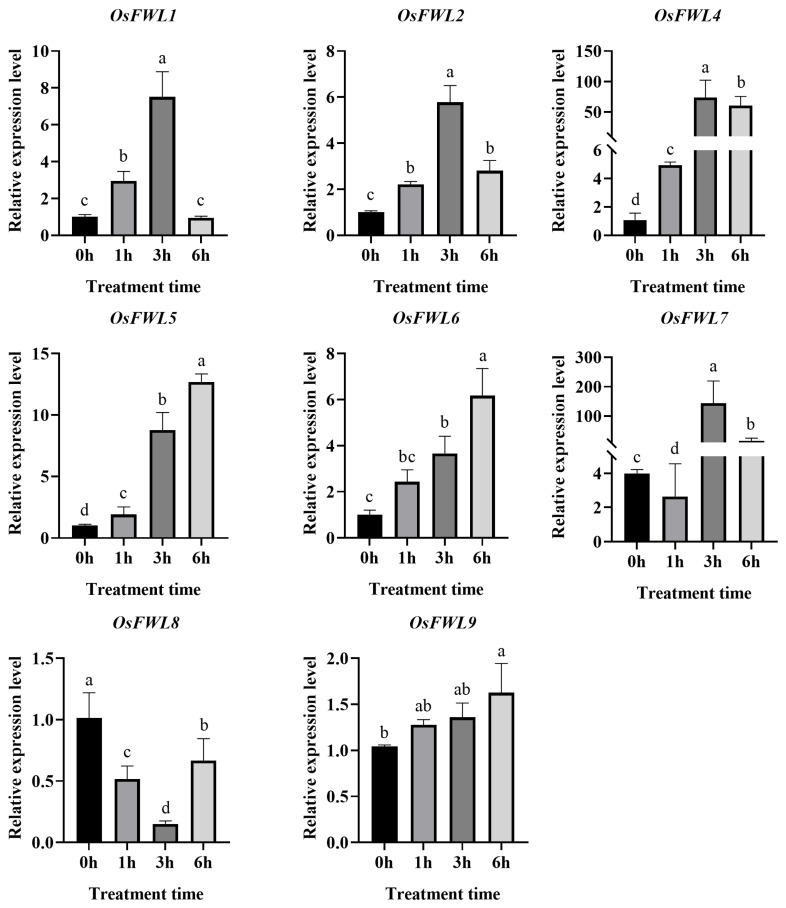
Expression analysis of selected *OsFWL* genes under arsenic stress. Data are presented as mean ± SD (*n* = 3). Different letters indicate significant differences at *p* < 0.05.

Members of the *OsFWL* gene family have previously been reported to respond to cadmium stress or to participate in cadmium transport. However, whether *OsFWL* genes are involved in responses to arsenic (As), another highly toxic metalloid, remains largely unclear. To investigate the transcriptional responses of *OsFWL* genes under arsenic stress, 14-day-old wild-type *Oryza sativa* cv. Nipponbare seedlings were treated with 40 µM As(III), and the expression levels of *OsFWL1*, *OsFWL2*, *OsFWL4*, *OsFWL5*, *OsFWL6*, *OsFWL7*, *OsFWL8* and *OsFWL9* were analyzed at 0, 1, 3, and 6 h after treatment. As shown in Figure 7, the expression patterns of most *OsFWL* genes under As stress were highly consistent with those observed under high-temperature, drought, and salt stress conditions. With the exception of *OsFWL8*, the remaining seven genes exhibited significant induction following As(III) treatment. Specifically, *OsFWL1*, *OsFWL2*, *OsFWL4* and *OsFWL7* reached peak expression levels at 3 h and subsequently declined, whereas *OsFWL5*, *OsFWL6* and *OsFWL9* displayed a sustained upward trend throughout the 6 h treatment period. In contrast, *OsFWL8* expression was markedly suppressed in response to As stress. These results suggest that most members of the *OsFWL* gene family are actively involved in the rice response to arsenic stress, although distinct expression dynamics exist among individual genes.

### 3.8. OsFWL8 Is a Novel Regulator of Arsenic Stress Response in Rice

Based on the qRT-PCR results described in Section 3.7, *OsFWL8*, whose biological function has not been previously reported, was selected for further functional analysis. To this end, *OsFWL8* overexpression and knockout lines were generated to determine whether *OsFWL8* participates in the rice response to arsenic stress. First, the spatial expression pattern of *OsFWL8* was examined by qRT-PCR in different tissues of the As-tolerant *japonica* rice cultivar Nipponbare, including roots, stems, and leaves at the seedling stage, as well as roots, stems, flag leaves, leaf sheaths, nodes, young panicles (1–10 cm), and mature panicles (>15 cm) at the booting stage. *OsFWL8* expression was predominantly detected in roots and stems, particularly during the seedling and reproductive stages, where transcript levels were significantly higher than those observed in other tissues, such as panicles (Figure S2).

**Figure 8:**
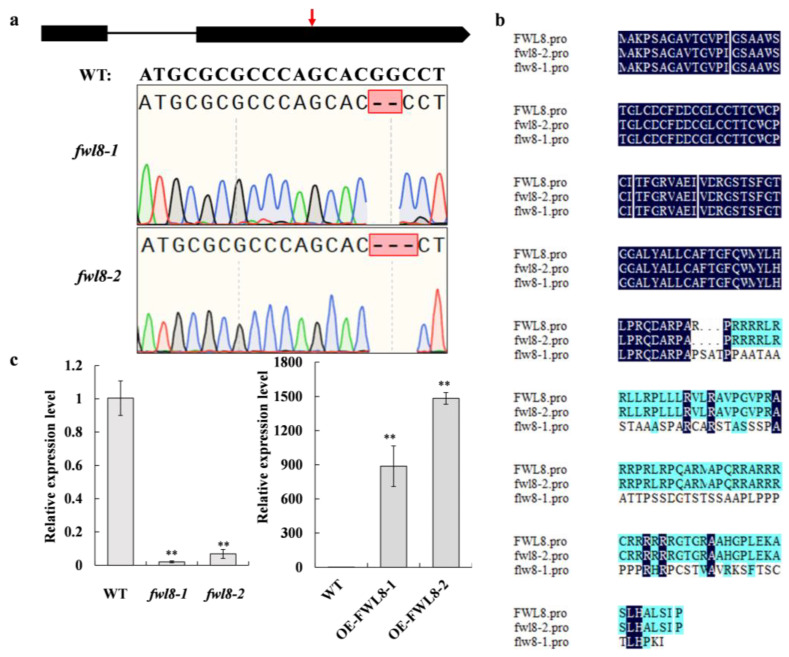
Molecular identification of *OsFWL8* knockout and overexpression lines. (**a**) CRISPR/Cas9 target sites in *OsFWL8*, with red arrows indicating mutation positions. (**b**) Amino acid sequence alignment of OsFWL8 in WT, *fwl8-1*, and *fwl8-2*. (**c**) qRT-PCR analysis of *OsFWL8* expression in knockout and overexpression lines. ** indicates significant differences at *p* < 0.01. Data represent three biological replicates.

To elucidate the biological function of *OsFWL8*, CRISPR/Cas9-mediated genome editing was employed to generate two independent homozygous knockout mutants, designated *fwl8-1* and *fwl8-2*. In *fwl8-1*, a two-base deletion (GG) in the second exon caused a frameshift mutation, resulting in disruption of the normal OsFWL8 protein sequence (Figure 8a,b). The *fwl8-2* mutant carried a three-base (CGG) deletion at the same position, leading to the loss of an arginine residue at the 90th amino acid position (Figure 8a,b). qRT-PCR analysis revealed that *OsFWL8* transcript levels were significantly reduced in both mutants compared with the wild type (Figure 8c). In parallel, the *OsFWL8* coding sequence was cloned into the pCAMBIA1300S vector to generate overexpression lines (OE-*OsFWL8-1* and OE-*OsFWL8-2*). qRT-PCR verification showed that *OsFWL8* expression levels in *OE-OsFWL8-1* and *OE-OsFWL8-2* plants were increased by approximately 887-fold and 1482-fold, respectively, relative to the wild type (Figure 8c).

**Figure 9:**
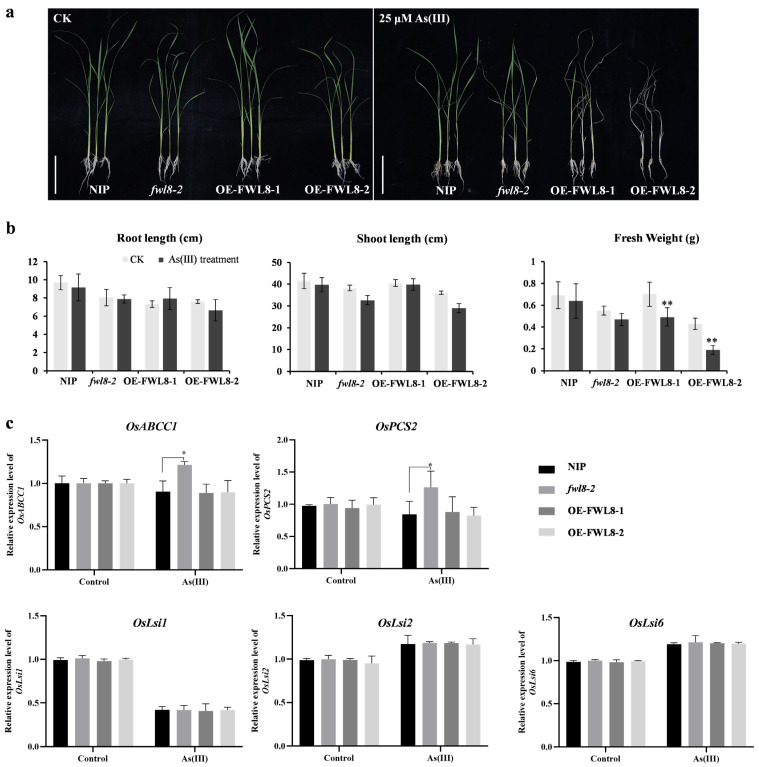
Phenotypic analysis of arsenic tolerance in *OsFWL8* knockout and overexpression lines. (**a**) Growth phenotypes under control and As(III) treatment (bar = 10 cm). (**b**) Statistical analysis of root length, shoot height, and fresh weight. (**c**) Expression analysis of arsenic-related genes. Data represent mean ± SD. * *p* < 0.05; ** *p* < 0.01.

To evaluate the role of *OsFWL8* in As tolerance, 21-day-old seedlings of Nipponbare (WT), *fwl8-2*, OE-*OsFWL8-1*, and OE-*OsFWL8-2* were grown in nutrient solution supplemented with 0 or 25 µM As(III) for 6 days, with 12 biological replicates per line. After one day of arsenic exposure, newly emerged leaves in all lines exhibited inward rolling. By the second day, leaves of WT and *fwl8-2* partially recovered, whereas those of the overexpression lines remained tightly curled. After three days, WT and *fwl8-2* plants resumed normal growth, while older leaves of OE-*OsFWL8-1* remained curled and those of OE-*OsFWL8-2* became completely desiccated. By day six, WT and *fwl8-2* plants displayed relatively healthy growth with only slight leaf-tip senescence, whereas all OE-*OsFWL8-2* plants had withered, and most OE-*OsFWL8-1* plants exhibited severe stem yellowing and failed to survive (Figure 9a). Fresh weight measurements further supported these observations. Compared with control conditions, fresh weight was reduced by approximately 8% in WT and *fwl8-2* plants, whereas reductions of about 30% and 56% were observed in OE-*OsFWL8-1* and OE-*OsFWL8-2* plants, respectively (Figure 9b). These results demonstrate that elevated *OsFWL8* expression markedly increases rice sensitivity to arsenic stress, particularly in the OE-*OsFWL8-2* line.

Given that high As(III) concentrations can induce osmotic stress and excessive arsenic accumulation in rice, we further examined the expression of key genes involved in arsenic detoxification and transport, including the phytochelatin synthase gene *OsPCS2* and arsenic transporter genes *OsABCC1*, *OsLsi1*, *OsLsi2*, and *OsLsi6*, in WT, *fwl8-1*, and OE-*OsFWL8* seedlings under control and arsenic treatment conditions. qRT-PCR analysis revealed no significant differences in the expression of *OsLsi1*, *OsLsi2*, or *OsLsi6* among the three genotypes. In contrast, *OsABCC1* and *OsPCS2* were significantly upregulated in the *OsFWL8* knockout mutants compared with WT and overexpression lines under arsenic stress (Figure 9c and Figure S3). Taken together, these findings demonstrate that *OsFWL8* plays a critical role in regulating arsenic tolerance in rice, with its overexpression markedly increasing arsenic sensitivity, potentially through modulation of downstream detoxification pathways.

## 4. Discussion

Fruit Weight 2.2 (FW2.2)-like (FWL) genes are evolutionarily conserved and widely distributed among plants, animals, and fungi. Previous studies have reported that the rice *FWL* gene family consists of eight members, designated *OsFWL1–OsFWL8* [21,31]. In contrast, through comprehensive sequence analysis and comparative genomics with *Arabidopsis thaliana*, we identified an additional and distinct member, *OsFWL9*, expanding the rice *FWL* family to nine genes (Figure 1). From the perspective of domain architecture, OsFWL9 contains only the PLAC8 superfamily domain, whereas the other eight OsFWL proteins harbor the canonical PLAC8 domain (Figure 2). Phylogenetic analysis involving *FWL* members from rice, *Arabidopsis*, maize, and soybean further revealed that *OsFWL9* clusters closely with *ZmFWL11*, suggesting a conserved evolutionary origin between these monocot species. In addition, protein sequence similarity and collinearity analysis indicated that *OsFWL1* is the ortholog of CNR1, consistent with previous reports [39].

Accumulating evidence suggests that the *FWL* gene family plays essential roles in plant growth, development, and stress adaptation. All FWL proteins contain the PLAC8 domain, and PLAC8-containing proteins in plants have been widely implicated in metal ion transport and organ size regulation [38]. For instance, overexpression of *TaCNR2* in wheat, *Arabidopsis*, and rice significantly enhances tolerance to cadmium (Cd), zinc (Zn), and manganese (Mn), while simultaneously reducing Cd accumulation in rice grains [39]. Similarly, SaPCR2 and BjPCR1 function as efflux transporters involved in Cd and Ca^2+^ tolerance, contributing to ion homeostasis in plants [40,41]. In rice, several *OsFWL* genes have been reported to regulate grain size and heavy metal transport; for example, *OsFWL1* and *OsFWL3* negatively regulate grain width and weight [28,39], while OsFWL4 may act directly as a Cd transporter and *OsFWL3–OsFWL7* enhance Cd resistance when expressed in yeast [25,26,27]. In the present study, *cis*-element analysis of *OsFWL* promoter regions revealed the presence of numerous stress-responsive regulatory elements (Figure 3). Consistently, qRT-PCR analyses demonstrated that most *OsFWL* genes respond to multiple abiotic stresses, including salt, drought, high temperature, and arsenic stress at the seedling stage (Figure 4, Figure 5, Figure 6, Figure 7 and Figure 9). These findings further support the notion that the *OsFWL* gene family plays a broad and important role in abiotic stress responses. Taken together, the dual involvement of *FWL* genes in seed size regulation and stress tolerance highlights their potential value in rice improvement. Manipulating *OsFWL* genes may provide a promising strategy to develop rice cultivars with both enhanced yield and improved tolerance to adverse environmental conditions, contributing to sustainable crop production and food security.

Our results revealed that most of *OsFWL* family members (*OsFWL1*, *OsFWL2*, *OsFWL4*-*9*) are actively involved in responses to high temperature, drought, salt, and arsenic stress. Notably, *OsFWL3* showed extremely low expression levels in rice seedlings and vegetative tissues, making it difficult to evaluate its role in abiotic stress responses (Figure S1). This observation is consistent with previous reports showing that *OsFWL3* is predominantly expressed in panicles at the heading stage and negatively regulates grain size, suggesting its specific involvement in reproductive development rather than stress adaptation [39]. Importantly, this study provides the first evidence that *OsFWL* family members respond to arsenic stress in rice. Functional analyses using transgenic lines revealed that *OsFWL8* overexpression markedly increased sensitivity to As(III). Notably, transcript levels of *OsABCC1* and *OsPCS2* were significantly elevated in *fwl8* mutants under arsenic stress (Figure 9). OsABCC1, a C-type ATP-binding cassette (ABC) transporter, restricts arsenic translocation to grains by sequestering arsenic into vacuoles in nodal phloem tissues, and loss of *OsABCC1* severely compromises arsenic tolerance in rice [18]. *OsPCS2* encodes phytochelatin synthase, which catalyzes phytochelatin production and facilitates arsenic sequestration into vacuoles, thereby mitigating arsenic toxicity [42,43,44,45]. The elevated expression of these genes in *fwl8* mutants may also demonstrate that the OsFWL8 gene plays a key role in the regulation of arsenic stress in rice (Figure S3). However, the precise molecular mechanism by which *OsFWL8* modulates arsenic detoxification pathways remains to be elucidated.

Overall, this study systematically identified and characterized nine *FWL* genes in rice and demonstrated that *OsFWLs* possess conserved motifs and promoter elements associated with stress responses, hormone signaling, and developmental regulation. The induction of *OsFWL* expression under drought, heat, salt, and arsenic stress, together with functional validation of *OsFWL8* in arsenic tolerance, highlights the multifaceted roles of this gene family. Collectively, our findings expand the current understanding of the rice *FWL* gene family and provide a valuable foundation for future investigations into their biological functions and regulatory mechanisms, particularly in the context of abiotic stress tolerance.

## Data Availability

The original contributions presented in this study are included in the article. Further inquiries can be directed to the corresponding authors.

## Supplementary Materials

The following supporting information can be downloaded at: https://www.mdpi.com/article/10.3390/plants15081146/s1, Figure S1: Tissue-specific expression analysis of *OsFWL3*. Data are presented as mean ± SD (n = 3). Different letters indicate significant differences (*p* < 0.05); Figure S2: Tissue-specific expression analysis of *OsFWL8*. 21d indicates seedlings at 21 days. Data represent three biological replicates; Figure S3: A schematic figure illustrating the proposed mechanism of *fwl8* in arsenic stress tolerance; Table S1: Annotation and amino acid sequences of 44 FWL/CNR proteins identified across 4 different plant species; Table S2: Primer sequences used in this paper.
