# Supplementary material for: Genome-Wide Identification of the FWL Gene Family in Rice Reveals Critical Roles in Abiotic Stress Response
Source: Plants (Basel). 2026 Apr 8;15(8):1146. doi: 10.3390/plants15081146 (PMC13118818; doi:10.3390/plants15081146)
Supplement: Supplementary file 1 [file plants-15-01146-s001.zip › Supplementary Figures.pdf]

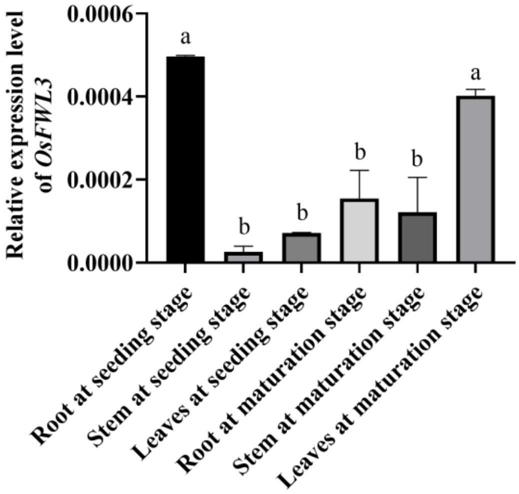

Figure S1. Tissue-specific expression analysis of *OsFWL3*. Data are presented as mean ± SD (n = 3). Different letters indicate significant differences ($p < 0.05$).

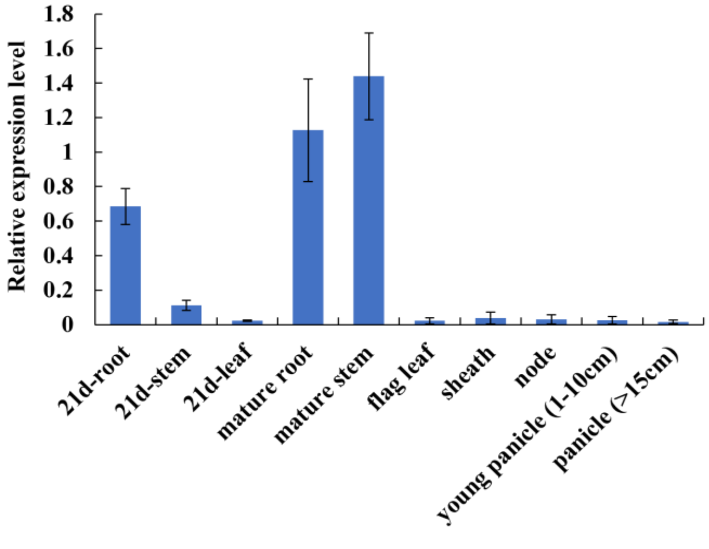

Figure S2. Tissue-specific expression analysis of *OsFWL8*. 21d indicates seedlings at 21 days. Data represent three biological replicates.

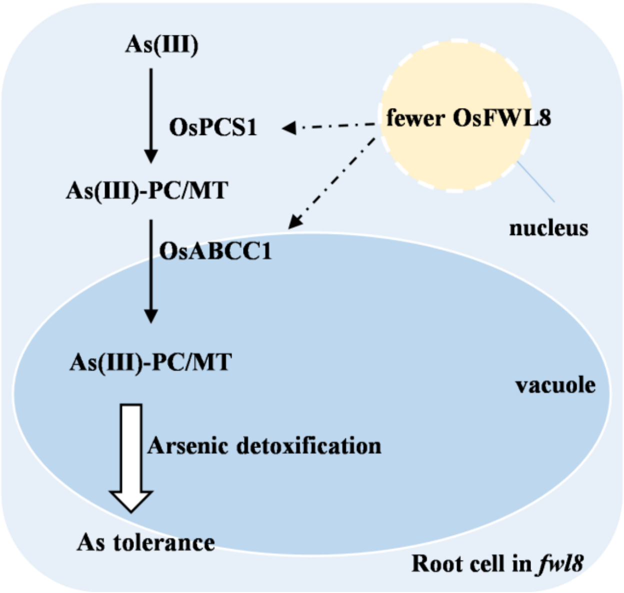

Figure S3. A schematic figure illustrating the proposed mechanism of *fwl8* in arsenic stress tolerance.
